# Biomarkers in Detection of Hepatitis C Virus Infection

**DOI:** 10.3390/pathogens13040331

**Published:** 2024-04-17

**Authors:** Jungreem Woo, Youkyung Choi

**Affiliations:** Division of Viral Hepatitis, National Center for HIV, Viral Hepatitis, STD and TB Prevention, US Centers for Disease Control and Prevention (CDC), Atlanta, GA 30329-4018, USA; qdq9@cdc.gov

**Keywords:** hepatitis C virus, detection markers for HCV infection, host biomarkers in acute and chronic hepatitis C, liver fibrosis/cirrhosis, hepatocellular carcinoma (HCC)

## Abstract

The hepatitis C virus (HCV) infection affects 58 million people worldwide. In the United States, the incidence rate of acute hepatitis C has doubled since 2014; during 2021, this increased to 5% from 2020. Acute hepatitis C is defined by any symptom of acute viral hepatitis plus either jaundice or elevated serum alanine aminotransferase (ALT) activity with the detection of HCV RNA, the anti-HCV antibody, or hepatitis C virus antigen(s). However, most patients with acute infection are asymptomatic. In addition, ALT activity and HCV RNA levels can fluctuate, and a delayed detection of the anti-HCV antibody can occur among some immunocompromised persons with HCV infection. The detection of specific biomarkers can be of great value in the early detection of HCV infection at an asymptomatic stage. The high rate of HCV replication (which is approximately 10^10^ to 10^12^ virions per day) and the lack of proofreading by the viral RNA polymerase leads to enormous genetic diversity, creating a major challenge for the host immune response. This broad genetic diversity contributes to the likelihood of developing chronic infection, thus leading to the development of cirrhosis and liver cancer. Direct-acting antiviral (DAA) therapies for HCV infection are highly effective with a cure rate of up to 99%. At the same time, many patients with HCV infection are unaware of their infection status because of the mostly asymptomatic nature of hepatitis C, so they remain undiagnosed until the liver damage has advanced. Molecular mechanisms induced by HCV have been intensely investigated to find biomarkers for diagnosing the acute and chronic phases of the infection. However, there are no clinically verified biomarkers for patients with hepatitis C. In this review, we discuss the biomarkers that can differentiate acute from chronic hepatitis C, and we summarize the current state of the literature on the useful biomarkers that are detectable during acute and chronic HCV infection, liver fibrosis/cirrhosis, and hepatocellular carcinoma (HCC).

## 1. Introduction

The hepatitis C virus (HCV) is a bloodborne pathogen that causes both acute and chronic hepatitis, ranging from mild illness to serious diseases including liver cirrhosis and hepatocellular carcinoma (HCC). In the United States, a total of 4848 acute HCV cases were reported during 2022, and the rate of reported cases remained the highest among persons aged 30 to 39 years (3.6 cases per 100,000 population) and 20 to 29 years (2.2 cases per 100,000 population), with injecting drug use as the principal risk factor [1]. Viral hepatitis surveillance indicates that the rate of reported acute hepatitis C cases are the highest among non-Hispanic American Indian/Alaska Native persons (2.9 cases per 100,000 population) compared with other race and ethnicity groups [1]. Acute hepatitis C is defined by the presence of any symptom of acute viral hepatitis plus either jaundice or elevated serum alanine aminotransferase (ALT) activity with the serologic presence of the anti-HCV antibody, HCV RNA, or the presence of hepatitis C viral antigen(s) (the HCV antigen) in the first 6 months after HCV infection [2]. In 2016, the case definition for acute HCV infection was expanded to include cases with positive HCV antibodies in the blood followed by a positive HCV antigen(s) test that is reported within the same year [3]. However, ALT levels can fluctuate, and a delayed seroconversion of anti-HCV antibodies may occur during acute HCV infection [4]. Most people with acute HCV infection are asymptomatic [5], but chronicity can lead to the development of cirrhosis and liver cancer. A more definitive means of diagnosing early infection could help to better identify the incidence and transmission of HCV infection, thereby improving diagnosis and surveillance. In this article, we review the current information regarding the biomarkers associated with acute and chronic HCV infection, liver fibrosis/cirrhosis, and HCC, as well as discuss how these biomarkers could be useful to provide a better tool for the detection of HCV infection. The scope of this review only includes an investigation of the biomarkers that are used to detect acute and chronic HCV infection in the adult population. HCV prevalence, access to screening, and treatment availability across different global regions are not discussed.

## 2. Natural History

Many persons with acute HCV infection develop chronic infection [6]. The mechanism for the development of chronic infection is not clear, but viral and host factors have been found to be involved in the development of persistent infection [7]. Based on the data from blood donors and community-acquired hepatitis C cases, 55% to 85% of HCV-infected persons develop chronic infection [8,9]. The prevalence of current HCV infection (i.e., detectable HCV RNA) is estimated from National Health and Nutrition Examination Survey (NHANES) data during 2017–2020, where 0.9% of adults were aged ≥ 18 years (approximately 2.2 million persons) [10]. This study also found that the current HCV infection was 6.4 times higher in persons aged 55–64 years to persons aged 18–40 years, 2.9 times higher in males than in females, and 5 times and 4.9 times higher in non-Hispanic White and non-Hispanic Black persons compared with persons of other races and ethnicities, respectively [10]. The high current HCV infection rate in persons aged 55–64 years may be related to the lower rate of spontaneous clearness among older adults when compared to persons aged 18–40 years [10].

## 3. Risk Factors for HCV

HCV is transmitted through exposure to infected blood. HCV was transmitted through blood transfusion before the commencement of blood screening in the 1990s [11]; however, the risk of HCV transmission through blood or blood products is extremely low now. In addition, hemodialysis [12], accidental needle stick [13], or unsafe therapeutic injection [14] cause HCV infection. Acute HCV infection has increased among persons aged 30 to 49 years [15,16]. Injection drug use is currently the major risk factor for HCV infection. In addition, HCV can be transmitted vertically from mother to child. The rate of HCV infection among the women of childbearing age increased 20% from 2016 through 2020, and it then declined 8% from 2020 through 2021 [17]. Hepatitis C testing is recommended for all adults aged ≥18 at least once and for pregnant women during every pregnancy [18].

## 4. HCV Replication

HCV is a member of the family Flaviviridae and the genus *Hepacivirus* [19,20]. The HCV genome is a single-stranded positive RNA of about 9600 nucleotides in length. HCV is classified into 8 different genotypes and 93 subtypes [21]. The replication of HCV starts with the initial attachment to hepatocytes. This process is mediated by the heparan sulfate proteoglycan syndecan-1 or syndecan-4 [22,23] or scavenger receptor class B type I (SR-BI) [24]. The entering of virus particles into hepatocytes occurs through the interactions between HCV envelope glycoproteins 1 and 2 (E1 and E2), SR-BI, tetraspanin CD81, claudin-1, and occludin [25,26,27,28]. HCV uptake occurs through endocytosis in a clathrin-dependent manner [29]. After fusion, HCV RNA is translated to produce viral proteins and to initiate the replication of viral RNA. HCV particles are released from the cells by a secretory pathway [30,31].

## 5. Pathogenesis of HCV Infection

Molecular mechanisms for the development of chronic infection have been intensely studied [32,33] but remain incompletely understood. While some studies have shown that clearance of infection is associated with the early appearance of broad neutralizing antibody responses during acute HCV infection and that a delayed induction of neutralizing antibodies in the late phase of infection leads to chronic HCV infection [34,35], other studies have reported no association between neutralizing antibodies and clearance of infection [36,37]. Other than antibody responses, cellular immune responses have a protective role in the outcome of acute HCV infection [38], where strong and poly-functional HCV-specific T cell responses are involved in the spontaneous clearance of infection, and that weak or narrower T cell responses lead to chronic infection [39]. The functional role of cytotoxic T lymphocyte (CTLs) responses shows that CD4+ T cells are critical both for limiting immune evasion and priming effector memory CTLs in resolving the acute HCV infections of humans and experimentally infected chimpanzees [40,41], whereas CD4+ and CD8+ T cells in chronic HCV infection are rapidly exhausted by the loss of interleukin-2 (IL-2) production and the increased expression of the programmed death 1 (PD-1) molecule, respectively [42,43]. After the introduction of first-generation, direct-acting antiviral (DAA) therapies in 2011, the restoration of immune cells like the natural killer cell function occurs through interferon-free therapy [44]. In addition, increased CD8+ T cell frequency and partial reversion in exhaustion markers on HCV-specific T cells have been found in patients with chronic hepatitis C [45,46,47]. However, others have shown that CD8+ T cell proliferation has a limited impact after the elimination of HCV [48]. These studies suggest that host genetic variation has an influence on HCV pathogenesis, including the development of chronicity and immune responses to the infection.

## 6. Detection Markers for HCV Infection

The development of HCV infection lasts up to six months and is considered an acute phase of infection [2,49]. Approximately 20% to 40% of people with acute hepatitis C clear the infection [50]. The factors associated with clearance are the female gender and people with symptomatic acute hepatitis C such as jaundice, fatigue, nausea, abdominal pain, and malaise [51,52]. Acute HCV infection can be diagnosed by the detection of HCV RNA and recent seroconversions to hepatitis C antibodies (anti-HCV). Approximately 1 to 2 weeks after exposure, HCV RNA is detectable by a nucleic acid test (NAT) [53]. Anti-HCV antibodies are detectable approximately 7 to 9 weeks after infection (ranging from 3 to 22 weeks) [53,54]. The currently recommended testing for HCV infection in the US consists of a Food and Drug Administration (FDA)-approved HCV antibody test followed by a test for HCV RNA if the antibody is positive [55,56]. Persons with the HCV antibody and detectable HCV RNA are considered to have a current HCV infection, and persons with HCV antibodies and undetectable HCV RNA have resolved or cleared HCV infection [56]. Anti-HCV testing, however, does not differentiate acute infection from chronic hepatitis infection. The HCV core antigen (HCV cAg) is released into the peripheral blood during viral assembly and can be detected around 12–15 days after infection. Presently, no HCV antigen test has been approved by the FDA. The presence of the HCV cAg is reported to be a sensitive and specific viral marker of current HCV infection [57,58]. As the two-step testing algorithm is an expensive and complex process, it may not be feasible in resource-limited settings [59]. HCV cAg assays can be considered as an alternative to NATs for the detection of HCV infection since it is more stable and easier to use [60,61]. Thus, concurrent testing for HCV cAg and anti-HCV could improve the early detection of HCV infection [62]. 

## 7. Host Biomarkers in Acute Hepatitis C

### 7.1. HCV Infection in Experimentally Infected Chimpanzees

Identifying the biomarkers for acute HCV infection remains a substantial challenge. Most acute cases are asymptomatic [63]. The only animal model of HCV infection, the chimpanzee model, improved the understanding of the pathogenic mechanism of HCV infection and provided valuable data on the innate and cellular immune responses induced during HCV infection [64,65]. As early events related to HCV infection are often unrecognized or missed in patients with acute hepatitis C, the liver tissues obtained from experimentally infected chimpanzees can allow us to analyze the events of early immune responses, as well as gene expression profiles, to identify the biomarkers of acute HCV infection. Moreover, the frequent collection of the liver tissue from the chimpanzees enabled us to analyze the kinetics of the host responses induced during acute HCV infection. Microarray analysis of the different expression levels in the liver tissue of chimpanzees with acute hepatitis C showed that clearance of infection is associated with a vigorous IFNα/β response, CD3e, and MlP-1α expression [66,67,68]. When chimpanzees with resolved infection are compared to the animals developing chronic HCV infection, the control of HCV replication follows after increased levels of the intrahepatic interferon-gamma (IFN-γ) and ALT levels, whereas the expression of those genes occurs about 2 weeks later when it comes to chimpanzees that have developed persistent infections [69]. Additionally, high levels of interleukin binding factor 3 (ILF3), cytotoxic granule-associated RNA binding protein (TIA1), and genes associated with the CD8+ T cell response are associated with acute self-limited infection [70]. Strong HCV-specific CD4+ and CD8+ T cell responses are observed among animals that clear the infection; in contrast, weaker T cell responses are observed among animals that progress to chronic HCV infection [71,72]. In our previous study, pattern-recognition receptor genes like RIG-1, TLR3, TLR7, and type I interferon genes were expressed in the liver as early as 7 days before the elevation of ALT activity [54]. The ALT activity coincided with the caspase 3/6 activity that was involved in the cell death signaling pathway that occurs before the activation of NK and T cells during acute HCV infection [54]. These studies provide information on the expression kinetics of the genes involved in the innate immune responses in the early phase of acute HCV infection.

### 7.2. HCV-Specific T Cell Responses

HCV-specific T cell responses play an important role in the outcome of acute HCV infection, where a vigorous and sustained T cell response targeted toward a broad range of HCV peptides is associated with HCV clearance, and where weak T cell responses lead to exhaustion or viral escape mutations that appear to contribute to persistent infection [73]. Persons with the same strain of HCV following blood exposure accidents such as needle stick injury have persistent HCV-specific CD4+ and CD8+ T-cell responses, while the humoral responses of HCV-specific antibodies are not detectable in many patients 18–20 years after recovery [63], thereby suggesting that HCV-specific CD4+ and CD8+ T cell responses can be the biomarkers for a prior HCV exposure and recovery. Activated HCV-specific CD8+ T cells express several markers like CD69, CD38, CD86, and HLA-DR during acute HCV infection [74,75,76,77,78]. Strong T cell responses to HCV nonstructural protein 3 (NS3), Th1 helper CD4+ T cell, and natural T cells expressing CD56 are associated with viral clearance in acute HCV infection [39,41,79,80,81]. T-cell exhaustion is one of the mechanisms that is thought to contribute to the progression of chronic HCV infection. PD-1 is a part of the CD28/cytotoxic T lymphocyte-associated antigen (CTLA-4) family, and it inhibits the T cell response by interfering with the T cell receptor signaling pathways [82]; however, its role in acute HCV infection remains controversial. The PD-1/programmed death ligand 1 (PD-L1) pathway is involved in HCV-specific CD8 exhaustion during HCV infection [83]. The activity of HCV-specific CD8 cells is restored by blocking the PD-1/PD-L1 pathway [83]. High expression levels of PD-1 on HCV-specific T cells during acute HCV infection are associated with progression to chronic HCV infection [84]. In contrast, PD-1 expression alone is insufficient to be a prognostic marker for chronic HCV infection [85]. In a recent study, HCV-specific CD4+ T cells were found to be phenotypically similar during early resolving and persistent infection, and they secreted similar levels of cytokines [86]. This study demonstrates that, after control of the viral infection, CD4+ T cells downregulate inhibitory receptors like PD-1 and CTLA-4 and differentiate into long-lived memory cells, whereas persisting viremia continue to activate T cell responses, as well as PD-1 and CTLA-4 expression, and block T cell differentiation until the cells disappear from the circulation [86]. This study indicates that the inhibitory receptor-mediated regulation of CD4+ T cells is not correlated with the outcome of infection in early HCV infection, and persistent HCV viremia leads to a sustained upregulation of PD-1 and CTLA-4. In addition, viral escape mutants from CD8+ T cell responses can lead to persistent infection in the acute phase of the infection [87,88]. These acute immune selection pressures can influence the outcome of HCV infection.

### 7.3. NK Cells

Natural killer (NK) cells are the innate immune cells providing antiviral immune responses by killing virus-infected cells and inducing T cell responses [89,90]. The activation of NK cells enhance cytotoxicity and IFN-γ production in acute HCV infection [91,92,93]. An increased frequency of CD56^bright^ NK cells is correlated with serum HCV core protein levels and normalization with spontaneous viral clearance [94]. In addition, the HCV core protein alters NK cell maturation and influences the outcome of acute infection. However, another study reported that the enhanced elimination of CD4+ T cells through increased NK cell number results in a loss of viral control and progression to chronicity in the acute phase of HCV infection [95]. These studies suggest that the immune responses are significantly variable in patients with acute HCV infection. 

### 7.4. Cytokines and Chemokines

Cytokines and chemokines are essential in antiviral responses during acute HCV infection. To identify potential biomarkers, various plasma- or serum-based cytokines and chemokines have been evaluated for their ability to separate viral clearance from persistent infection in patients with acute hepatitis C. The plasma IL-18, IP-10, and IFN-λ levels are elevated in the clearance of infection, and tumor necrosis factor (TNF)-α and the IL 10, B-lymphocyte stimulator/B activating factor (BLyS/BAFF) (the TNF-family cytokine involved in B-cell proliferation) are increased in non-resolved patients [96,97,98,99,100]. Patients with acute HCV infection and high baseline IP-10 levels are associated with a failure to spontaneously clear HCV [101]. In another study, the levels of IP-10 were increased when the levels of ALT and HCV RNA were elevated, and high HCV RNA levels were accompanied by elevated MIP-1β expression [99]. The resolution of acute HCV infection is linked to IFN-λ1 (IL-29) production [99,102]. The N-terminal truncated form of CXCL10, generated by the protease dipeptidylpeptidase 4 (a potent chemoattractant for antiviral T-cells and NK-cells), is associated with a failure to achieve the spontaneous clearance of acute HCV infection [103].

### 7.5. Other Biomarkers

HCV IgG antibody avidity, the binding strength of the HCV-specific IgG antibody to antigens, differentiates the recent HCV infections from chronic HCV infections [104,105,106]. HCV IgG antibody avidity is also used as a biomarker to estimate the population-level incidence of HCV infection where the mean duration of a recent infection is calculated to estimate the HCV infection incidence among high-risk populations [107,108]. Single nucleotide polymorphisms (SNPs) in the *IL28B* gene (*rs12979860*) encoding the type III interferon 3 (IFN-λ3) enhance the spontaneous clearance of HCV infection [109]. The combination of the serum levels of IP-10 and SNP in *IL28B* [110] and *CTLA4* polymorphisms [111] are found in the spontaneous clearance of HCV infection. HCV is bound to plasma lipoproteins and circulates as an infectious lipoviral particle (LVP) [112]. Testing for the maximum concentration of LVP (Maxi-LVP) as a biomarker shows that the low median value of the maxi-LVP is independently associated with spontaneous clearance [113]. A high level of plasma apolipoprotein C-III is connected to the spontaneous resolution of HCV infection [114]. Lower expressions of liver-associated lipoproteins (Apo B, Apo D, Apo H, and α1-AT) can distinguish HCV-infected patients from HAV- and HBV-infected individuals [115]. A comprehensive analysis of the host genetic factors contributing to the variability in HCV infection outcomes are not discussed in this section.

### 7.6. microRNAs

MicroRNAs (miRNAs), i.e., non-coding small RNAs, participate in diverse pathological and physiological processes by binding to the complementary regions of targeted gene expression [116,117,118]. Various miRNAs are differently expressed during acute HCV infection. For example, miR-122, a liver-specific miRNA, constitutes 72% of total liver miRNA [119]. Our study of the relationship between the intrahepatic expression of miR-122, HCV replication, and liver damage, as measured by the serum ALT elevation during the acute phase of HCV infection, found that hepatic miR-122 levels are inversely correlated with HCV RNA titers in the liver, and the serum levels of miR-122 expression are positively correlated with serum HCV RNA and ALT activity during acute HCV infection [120]. The hepatic expression of miR-122 and miR-126 correlates with serum HCV RNA, and miR-136 and miR-122 correlate with steatosis [121]. Hepatic miR-122 levels are up-regulated in women relative to men, and they are associated with portal inflammation and HCV genotype 3 [121,122]. Circulating miR-20a and miR-92a levels are unchanged in HCV-infected patients, who progress from acute to chronic infection, and miR-92a expression is reduced in acute-to-resolved individuals [123].

### 7.7. Mitochondrial DNA

Additionally, serum mitochondrial DNA (mtDNA) genetic diversity induced by HCV infection can be used to discriminate recent from past infections, where the difference in mtDNA heterogenicity is measured by the degree of nucleotide diversity in HCV hypervariable region 1, which could be used to separate between acute and chronic HCV infection [124]. The relevant biomarkers for acute HCV reported in these studies are summarized in Table 1.

## 8. Biomarkers in Chronic Hepatitis C Virus Infection

Most people with hepatitis C develop persistent infection, and the mechanism for the development of chronic infection remains incompletely understood; however, viral innate immune evasion and defective adaptive immunity could be the causes of the progression of acute HCV infection to viral persistence [125,126].

1. Chronic HCV infection in experimentally infected chimpanzees

Chronic HCV infection in chimpanzees induces lower HCV-specific immune responses and milder liver damage [127,128]. Chronic HCV infection induces increased expression of Interferon-stimulated genes (ISG) and steatosis genes in the livers of chimpanzees [129,130]. However, the levels of ISG expression in chronic HCV infection are lower than those induced by acute HCV infection. High levels of indoleamine 2,3-dioxygenase (IDO), CTLA-4, and PD-1 expression are found in chimpanzees with chronic HCV infection [131]. In the case of HCV reinfection in chimpanzees, the duration of viremia is short and rarely persistent [132]. The resolution of the subsequent HCV infection is associated with rapid type I IFNα and IFNγ responses [133,134]. In addition, memory CD8+ T lymphocytes are required to rapidly terminate HCV replication upon re-exposure to the virus [135].

2. HCV-specific T cells

Inefficient cellular immune responses contribute to viral persistence in chronic HCV infection. HCV-specific CD8+ T cells derived from chronically infected patients display an impairment of proliferative, cytokine, and cytotoxic effector functions than those from recovered patients [136,137]. In addition, after the first 6 months of HCV infection, T cell responses decline and there is a loss of antigen recognition over time in patients who develop persistent HCV infection [138]. This observation may be associated with viral escape mutation because viral quasispecies diversification occurs throughout infection. The induction of suppressor T cells and dendritic cell dysfunction are found to be associated with chronic HCV infection [139,140]. Continuous antigen exposure during chronic HCV infection induces the loss of the effector function or exhausted HCV-specific CD8+ T cells [112]. Exhausted CD8+ T cells were first described in lymphocytic choriomeningitis virus (LCMV) infection, in which the expression of PD-1 is upregulated by the exhausted T cells [141]. PD-1 in CD8 T cells regulates the glycolytic and mitochondrial metabolism, suggesting that T cell exhaustion may be involved in the metabolic pathways [142]. HCV-specific CD4+ T cells also rapidly disappear and impair cytokine production with the expression of PD-1 and CTLA-4 expression during chronic infection [143,144]. The macrophage-related serum biomarker soluble CD163 (sCD163) has been shown to differentiate mild liver fibrosis from the cirrhosis in patients with chronic hepatitis C [145]. The level of sCD163 increases with the disease severity [146], and it declines with successful direct-acting antiviral therapy [147].

3. Cytokines and chemokines

Cytokines and chemokines play an important role against HCV infection, and the kinetics of their expression have been intensely investigated during chronic HCV infection. Helper T cells type 1 (Th1) promote cellular immune responses and produce cytokines such as IFN-γ and IL-2, while the Th2 cells that produce IL-4 and IL-10 are involved in humoral immune responses [148]. The Th1 and Th2 cytokine balance in the sera and liver tissues of patients with chronic hepatitis C contributes to the pathogenesis of the chronic phase of the infection. Cytokine responses are either shifted to Th1 (IFNγ, IL-18) [149,150,151] or Th2 (IL-10) expression [152], whereas lower Th1 cytokine expression [153] and decreased intrahepatic IL-10 expression [154] are associated with persistent infection and severe fibrosis during chronic HCV infection, respectively. IL-17 secreting T cells (Th17), another helper T cell subset, have roles in the host immunity against intracellular pathogens and chronic inflammatory conditions like rheumatoid arthritis, psoriasis, and multiple sclerosis [155]. HCV-specific Th17 cells are suppressed by virus-induced TGF-β [156], and IL-17 and IL-22-producing T cells are enriched in the liver of patients with chronic HCV infection [157]. Serum IL-4, IL-10 [158], and IL-18 [159] levels are higher in patients with chronic HCV infection. Serum IL-26, a member of the IL-10 cytokine family, levels are elevated in chronic hepatitis C patients with severe liver inflammation, where IL-26 upregulates TNF-related apoptosis-inducing ligand (TRAIL) expression on NK cells and then kills HCV-infected hepatoma cells [160]. Also, IL-26 induces the expression of the antiviral cytokines IFN-β and IFN-γ, as well as the proinflammatory cytokines IL-1β and TNF-α, by NK cells [160].

4. microRNAs

Intracellular miRNAs are secreted from cells through the exosomes and microvesicles that are present during cell death and which can be detected in plasma and sera [161]. These circulating miRNAs have potential as non-invasive biomarkers. Several studies have demonstrated an association between circulating miR-122 levels and liver injuries such as acute liver failure, as well as in cirrhosis induced by chronic HCV infection [162,163,164,165,166,167]. However, other studies have shown no association between serum miR-122 levels and chronic HCV infection [168], or even decreased serum miR-122 expression in chronic HCV patients [169]. Meta-analyses have indicated that circulating miRNA-122 has a relatively high diagnostic value for chronic HCV infection [170]. A combination of several microRNAs (miR-122, miR-126, miR-129, miR-199a, miR-155, miR-203a, miR-221, and miR-223) have demonstrated use as non-invasive biomarkers for staging HCV-associated liver fibrosis [171]. Other miRNAs, serum miR-21 [172], hepatic miR-146a-5p [173], miR-16, miR-193b, miR-199a, miR-222, and miR-324 in peripheral blood mononuclear cells (PBMC) [174] have also been found to be elevated in patients with chronic HCV. Serum miRNA-196a is lower in chronic HCV patients [175]. The serum levels of miR-20a, miR-215-5p, miR-483-5p, miR-193b-3p, miR-34a-5p, miR-885-5p, miR-26b-5p, and miR-197-3p are associated with liver fibrosis [123,176]. Extracellular vesicles (EVs) are major players in cell–cell communication, contain various microRNAs and mRNAs, and change pathophysiological states by HCV infection [177]. Among circulating EV-associated miRNAs, the upregulation of miR-122 and downregulation of miR-200b and miR-192, as well as miR-200b, miR-92a, and miR-150, are associated with the early stages of liver fibrosis [178]. The relevant biomarkers for chronic HCV infection reported in these studies are summarized in Table 2.

## 9. Biomarkers for Liver Fibrosis/Cirrhosis

Chronic HCV infection often leads to liver fibrosis and cirrhosis. There is a great need for non-invasive tests to diagnose and monitor liver fibrosis in patients with chronic HCV infection. Although liver biopsy has been a standard for the assessment of liver fibrosis, the liver biopsy procedure is costly and may involve complications. Non-invasive serum biomarkers have been investigated to replace liver biopsies, where a combination of several serum markers including alpha2-macroglobulin, haptoglobin, apolipoprotein A1, gamma-glutamyl transpeptidase, and bilirubin, show positive predictive values to liver fibrosis in patients with hepatitis C [179]. These serum markers, which are marketed as FibroTest and FibroScan, use noninvasive methods for the measuring of liver fibrosis biomarkers. The diagnostic test accuracy of FibroTest and FibroScan indicate that these tests could identify HCV-related fibrosis/cirrhosis, but they have decreased accuracy for earlier stages of fibrosis [180]. When FibroTest is compared to a liver biopsy, FibroTest has a better 5-year prognostic value than biopsy estimates regardless of the treatment, and it also has risk factors that are similar to that of liver biopsies [181]. Other non-invasive markers like aspartate aminotransferase (AST) to the platelet ratio index (APRI) [182] and FIB-4 index, which combines standard biochemical values (platelets, ALT, and AST) and age [183], enable the identification of chronic hepatitis C patients with fibrosis and cirrhosis. The relevant biomarkers for liver fibrosis/cirrhosis are summarized in Table 3.

## 10. Biomarkers for HCC

HCC is the most common type of primary liver cancer and the leading cause of death among patients with cirrhosis induced by nonalcoholic fatty liver disease, alcohol-related liver disease, and HCV infection [184]. Different serum or tissue biomarkers have been studied, but the clinical usage has not been widely accepted. Due to the poor utilization of surveillance for patients with cirrhosis and a lack of risk-based strategies, most patients are diagnosed at late stages [185]. Alpha-fetoprotein (AFP) is the most commonly used biomarker in the early detection of HCC [186]. The role of the AFP in HCC surveillance is contradictory. The association between AFP and chronic HCV infections indicates that high levels of serum AFP in chronic HCV patients are correlated with lower serum albumin levels and advanced fibrosis [187], whereas the elevated levels of serum AFP in patients with chronic hepatitis C is independently associated with stage III/IV hepatic fibrosis [188]. The serum levels of AFP-L3 (i.e., *Lens culinaris* agglutinin-reactive AFP, a fucosylated glycoform of AFP) and Des-gamma carboxyprothrombin (DCP) can serve as biomarkers for the early detection of HCC in patients with hepatitis or liver cirrhosis [189,190]. Circulating EVs excreted by cells are present in the early stage of the disease and persistent in all disease stages [191]. The EVs secreted by tumor cells have been evaluated as a biomarker using chip-based technology to show their potential for the noninvasive early detection of HCC [192]. In addition, Golgi protein 73 (GP73) (a 73-kDa human resident Golgi membrane protein) [193] and Golgi phosphoprotein 2 levels (GOLPH2) [194] can also serve as serum biomarkers for HCC. The biomarkers for HCC are summarized in Table 4.

## 11. Conclusions and Future Directions

The biomarkers for acute and chronic HCV infection have been investigated over the past few decades. Identifying the biomarkers of HCV infection is difficult because the quasispecies of the virus exists even within a single infected individual [195,196], which affects the infection outcome and presents difficulty in identifying the acute state. Identifying host biomarkers such as the early immune response can also vary between individuals. More definitive means of diagnosing early infection are needed. Acute HCV infections rarely cause clinical symptoms, and most acute infections become chronic leading to future cirrhosis and liver cancer for many [197,198]. During the early acute phase of the infection, markers of the innate immune responses include interferon (IFN) alpha responses and natural killer cells, as well as the rapid induction of cytokines and chemokines [54,67,68,69]. In addition, the vigor and quality of T cell responses are important to determine the outcome of acute HCV infection [76,138]. Despite the activation of innate immune responses and recruitment of HCV-specific T cell responses, infection becomes chronic for most patients with hepatitis C. The molecular mechanism of the development of chronic HCV infection is still unclear. Highly effective DAA therapy has revolutionized the treatment of chronic HCV infection [199,200]. After DAA therapy, both innate and adaptive immune homeostasis are restored [45,201,202]. However, DAA therapy does not eliminate the risk factors for HCC development. Further studies would provide a better understanding of the molecular mechanisms induced by HCV-induced HCC, as well as identify biomarkers for the early detection of cancer risk. The recent development of high-throughput single-cell RNA sequencing would allow us to measure cell types of innate and cellular immune responses in patients with HCV infection. For example, early transcriptional signatures related to HCV-specific primary human CD8+ T cells in chronic versus acute infection in humans have shown that genetic diversity, variability, differences in age and comorbidities, and environmental factors influence both the immune responses and outcome of infection [203]. These technological advances would allow us to identify the differences in the human immune system at a global scale by analyzing the variations in specific cell populations, as well as by taking proteins into account. System biology can help us to understand the immune response mechanism to acute and chronic HCV infection for better diagnoses and management of hepatitis C.

## Figures and Tables

**Table 1:** The biomarkers in acute HCV infection.

Class	Biomarker	Outcome	Reference
HCV infection in chimpanzees	↑ RIG-1, TLR3, TLR7, and type I interferon genes	Elevation of ALT activity	[54]
	↑ Caspase 3/6 activity	Activation of NK and T cells	[54]
	↑ IFNα/β response, CD3e, and MlP-1α expression	Spontaneous clearance	[66,67,68]
	↑ Intrahepatic IFN-γ and ALT activities	Spontaneous clearance	[69]
	↑ ILF3, TIA1, and genes associated with CD8+ T cell response	Spontaneous clearance	[70]
	↑ HCV-specific CD4+ and CD8+ T cell responses	Spontaneous clearance	[71,72]
HCV-specific T cell responses	↑ CD69, CD38, CD86, and HLA-DR expression on HCV-specific CD8+ T cells	Acute HCV infection	[74,75,76,77,78]
	↑ T cell responses to NS3, Th1 helper CD4+ T cell, and natural T cells expressing CD56	Viral clearance	[39,41,79,80,81]
	↑ PD-1 on HCV-specific T cells	Persistent infection	[83,84]
	↑ Viral escape mutants from CD8+ T cell responses	Persistent infection	[87,88]
NK cells	↑ NK cell activity	Spontaneous clearance	[91,92,93]
	↑ CD56bright NK cells	Spontaneous clearance	[94]
	↓ CD4+ T cells by ↑ NK cell numbers	Persistent infection	[95]
Cytokines and chemokines	↑ Plasma IL-18, IL-29, IP-10, and IFN-λ	Spontaneous clearance	[96,97,99,101]
	↑ Plasma TNF-α, IL-10, and BLyS/BAFF	Persistent infection	[97,100]
	↑ IP-10	Persistent infection	[101,103]
	↑ IL-29	Spontaneous clearance	[99,102]
Other biomarkers	↓ HCV IgG antibody avidity	Acute HCV infection	[104,105,106]
	↑ HCV IgG antibody avidity	Chronic HCV infection	[104,105,106]
	IL28B polymorphism	Spontaneous clearance	[109]
	serum IP-10 levels/IL28B polymorphisms	Spontaneous clearance	[110]
	CTLA4 polymorphisms	Spontaneous clearance	[111]
	LVP in early infection	Persistent infection	[112,113]
	↑ Plasma apolipoprotein C-III	Spontaneous clearance	[114]
	↓ Apo B, Apo D, Apo H, and α1-AT	Distinguish HCV from HAV and HBV	[115]
MicroRNAs	Hepatic miR-122	Inverse correlation with liver HCV RNA titer	[120,121,122]
	Serum miR-122	Positive correlation with serum HCV RNA titer	[120]
	Hepatic miR-122 and miR-126	Serum HCV load	[121]
	Hepatic miR-136 and miR-122	Steatosis	[121]
	↓ Plasma miR-92a	Viral clearance	[123]
Mitochondrial DNA (mtDNA)	nucleotide diversity	Distinguish acute from chronic HCV infection	[124]

↑, high levels of expression, ↓, low levels of expression.

**Table 2:** Biomarkers in chronic HCV infection.

Class	Biomarker	Outcome	Reference
HCV infection in chimpanzees	↑ OAS, MxA, ISG15, ISG20, IRF7, STAT1α/β,IFIT4, and ADAR1	Chronic HCV infection	[129,130]
	↑ IDO, CTLA-4, and PD-1	Chronic HCV infection	[131]
HCV-specific T cells	↓ HCV-specific CD8+ T cells	Chronic HCV infection	[136,137,138]
	Dysfunction of dendritic cells	Chronic HCV infection	[139,140]
	↓ HCV-specific CD4+ T cells with PD-1 and CTLA-4 expression	Chronic HCV infection	[143,144]
	soluble CD163 and soluble mannose receptor (sMR)	Cirrhosis induced by chronic HCV	[145]
Cytokines and chemokines	↑ Th1 cytokine (IFNg, IL-18)	Chronic HCV infection	[149,150,151]
	↑ Th2 (IL-10)	Chronic HCV infection	[152]
	↓ Th1 cytokine	Chronic HCV infection	[153]
	↓ Hepatic IL-10	Chronic HCV infection	[154]
	↓ HCV-specific Th17 cells by HCV-induced TGF-b	Chronic HCV infection	[156]
	↑ Hepatic IL-17 and IL-22-producing T cells	Chronic HCV infection	[157]
	↑ Serum IL-4, IL-10, and IL-18	Chronic HCV infection	[158,159]
	↑ Serum IL-26	Chronic HCV infection with severe liver inflammation	[160]
MicroRNAs	↑ Serum miR-122 with liver injuries	Chronic HCV infection	[162,163,164,165,166,167]
	↓ Serum miR-122	Chronic HCV infection	[169]
	Serum miRNA-122 as diagnostic value	Chronic HCV infection	[170]
	Combination of miR-122, miR-126, miR-129, miR-199a, miR-155, miR-203a, miR-221, and miR-223	Liver fibrosis	[171]
	↑ Serum miR-21	Chronic HCV infection	[172]
	↑ Hepatic miR-146a-5p	Chronic HCV infection	[173]
	↑ PBMC miR-16, miR-193b, miR-199a, miR-222, and miR-324	Chronic HCV infection	[174]
	↓ Serum miR-196a	Chronic HCV infection	[175]
	Combination of miR-20a, miR-215-5p, miR-483-5p, miR-193b-3p, miR-34a-5p, miR-885-5p, miR-26b-5p, and miR-197-3p	HCV-induced liver fibrosis	[123,176]
Extracellular vesicles (EV) associated miRNA	↑ miR-122, ↓miR-192, miR-200b, miR-150, and miR-92a	Early-stage fibrosis	[178]

↑, high levels of expression, ↓, low levels of expression.

**Table 3:** Biomarkers for liver fibrosis/cirrhosis induced by HCV infection.

Class	Biomarker	Outcome	Reference
FibroTest/FibroScan	Combination of several serum markers (alpha2-macroglobulin, haptoglobin, apolipoprotein A1, gamma-glutamyl transpeptidase, and bilirubin)	Non-invasive marker for HCV-induced fibrosis	[179,180,181]
APRI	AST to platelet ratio index	Non-invasive marker for HCV-induced fibrosis/cirrhosis	[182]
FIB-4 index	Combination of platelets, ALT, AST, and age	HCV-induced fibrosis and cirrhosis	[183]

**Table 4:** Biomarkers of HCV-related hepatocellular carcinoma (HCC).

Class	Biomarker	Outcome	Reference
Alpha-fetoprotein (AFP)	Serum AFP	Early detection of HCC	[186]
	Serum AFP-L3 and DCP	Early detection of HCC	[189,190]
Extracellular vesicles (EV)	EVs secreted by HCC	Early detection of HCC	[191,192]
Golgi proteins (GP)	↑ Serum GP73	HCC	[193]
	↑ Serum GOLPH2	HCC	[194]

↑, high levels of expression, Des-gamma carboxyprothrombin (DCP), Golgi phosphoprotein 2 (GOLPH2).
